# Improving Translation and Cultural Appropriateness of Spanish‐Language Consent Materials for Biobanks

**DOI:** 10.1002/eahr.500028

**Published:** 2019-09-20

**Authors:** Kathleen M. Brelsford, Ernesto Ruiz, Catherine M. Hammack, Laura M. Beskow

**Affiliations:** ^1^ Research assistant professor in the Center for Biomedical Ethics & Society at Vanderbilt University Medical Center; ^2^ Postdoctoral fellow in the Department of Family and Community Medicine at Meharry Medical College; ^3^ Associate in health policy in the Center for Biomedical Ethics & Society at Vanderbilt University Medical Center; ^4^ Professor and the Ann Geddes Stahlman chair in medical ethics in the Center for Biomedical Ethics & Society at Vanderbilt University Medical Center.

**Keywords:** human subjects research, biobanks, consent forms, translation

## Abstract

A growing proportion of prospective research participants in the United States speak limited or no English. We conducted cognitive interviews with native Spanish speakers to test Spanish‐language translations of simplified and traditional biobank consent forms. Comprehension was generally high and did not differ by form. Most of those who received the simplified form felt it contained the right amount of information, compared with fewer than half of those who received the traditional form. Qualitative results allowed us to identify overarching issues related to tone, formality, and voice that may affect prospective participants’ trust and willingness to participate. Certain characteristics of written Spanish are seemingly at odds with recommended actions to simplify consent forms; thus, even when significant empirical effort has been expended to develop simplified consent materials in English, additional work is needed to ensure the accuracy, comprehensibility, and cultural‐congruence of Spanish‐language translations.

**A** growing proportion of prospective research participants speak limited or no English.^1^ In the United States, Spanish is by far the most common language spoken at home after English; as of 2016, approximately 38 million U.S. residents (roughly 13% of the population) speak Spanish at home.^2^ Among them, nearly 10 million do not speak English well or at all.^3^ Although research consent forms are routinely translated from English into Spanish, cognitive testing is not necessarily conducted to ensure the accuracy, comprehensibility, and cultural‐congruence of these materials.^4^

The subjective nature of language and the inability to directly translate all relevant ideas across languages provides ample opportunity for concepts—even those rigorously tested in English—to become altered or altogether lost in translation.^5^ Translated materials may inadvertently omit key information, change the substantive meaning of the information, or introduce more technical language.^6^ Moreover, the specific tone, sequence of information in, and structure of standard research consent materials may not easily translate across cultural and linguistic variants.^7^ Translations that fail to attend to these variations can lead to embarrassment, confusion, and misperceptions on the part of potential participants, which can in turn promote distrust.^8^

Federal regulations governing research with humans require that consent materials be presented “in language understandable to the subject,”^9^ yet many forms continue to be written in complex language that hinders comprehension.^10^ Efforts have been made to devise and test simpler consent materials empirically, yet these are often limited to the development of English‐language materials.^11^ The regulations also require that consent materials contain the information that a reasonable person would want in order to make an informed decision about participating.^12^ Although several studies have examined the types and depth of information English‐language speakers want to receive in consent materials,^13^ it is unclear whether Spanish‐language speakers regard similar information to be vital to making decisions about participating in research.

Cognitive interviews provide a valuable tool to assess and improve the clarity and comprehensibility of consent materials,^14^ as well as to ensure that their tone, formality, and content are congruent with linguistic and cultural norms.^15^ Such interviews can also help ensure that consent materials include the types and amount of detail that Spanish‐language speakers want to know.^16^ In this study, we conducted cognitive interviews with 39 native Spanish speakers in Los Angeles and New York City to test Spanish‐language translations of simplified and traditional consent forms for a hypothetical biobank. We used open‐ended questions to identify aspects of the consent materials participants found reassuring, concerning, or confusing. We also used a 15‐question comprehension quiz to assess understanding of key concepts.

## Study Methods

**W**e hired a professional research firm with a field site in each location to assist with recruitment. Prospective participants were screened by telephone to determine eligibility, which included being at least 18 years of age, having emigrated to the U.S. at age five or older, speaking Spanish as a native language, speaking Spanish as the primary language in the household, and being able to read Spanish. Among eligible participants, we aimed to maximize diversity in terms of age, education level, and gender. Given broad dialectic variation in Spanish‐language usage and meaning, we also sought to interview Spanish speakers from different regions.

Interviews were conducted in person between December 2016 and January 2017 by a native Spanish speaker (ER). The study sample was demographically diverse and comprised native Spanish speakers who speak 12 regional varieties of Spanish (North America, South America, Central America, and the Caribbean) (see table 1). Materials participants received included a six‐page traditional form explicitly designed to closely resemble biobank consent forms in actual use, a three‐page simplified consent form, and a 15‐question comprehension assessment with accompanying explanations of the correct answers (available from the authors upon request). All three consent‐related documents were empirically developed in English^17^ and then translated into Spanish through a rigorous process.^18^ Five Spanish speakers were involved in the translation process. Two collaborated to draft the initial forward translations. For each translation, a third team member,

Many characteristics of written Spanish are seemingly at odds with recommended actions to simplify consent forms.

blinded from both the original and Spanish documents, then completed a back translation. Two team members used the compare feature in Word to identify differences between the original document and back translation, which were then compared against the Spanish translation. Additional revisions were then made.

We used a nonrandom strategy to assign interviewees to read either the simplified or traditional biobank consent form to maximize demographic balance on key factors (such as education level, country of origin, gender, and age). At the beginning of the interview, participants read the entire form they were assigned and then completed the quiz. Using a semistructured interview guide (available upon request), we then walked through the form with participants, section by section. Within each section, we asked participants three separate questions to explore areas they found unclear or awkward, concerning, or reassuring. After participants had an opportunity to consider the consent form in depth, we asked them to rate their satisfaction with the amount of information in the form and their willingness to participate in the hypothetical biobank in order to get a sense of how, in the end, they weighed and balanced the considerations they identified and the factors they discussed. All interviews were audio recorded and lasted an average of 60 minutes. Participants received $90 for their time. (Institutional review boards at Duke University and Vanderbilt University Medical Center deemed this research exempt under 45 CFR 46.101[b][2] [2009]).

We used an applied thematic approach to code, analyze, and interpret qualitative data.^19^ The professionally transcribed Spanish‐language interviews were first uploaded into the qualitative coding software NVivo 11. One author (ER) applied mutually exclusive structural codes to delineate text by interview question and then applied secondary structural codes to identify major domains within each code.^20^ Two authors (ER and KMB) reviewed five transcripts to identify an initial set of content codes; they then independently applied codes to three additional transcripts, reviewed discrepancies, and made further revisions to the codebook. They continued this iterative process until they achieved at least 80% intercoder agreement.^21^ The primary coder (ER) then coded the remaining transcripts, and the secondary coder reviewed every fourth transcript to ensure a high degree of intercoder agreement. All steps were completed using the Spanish‐language transcripts. (In an online supplement, we provide all directly quoted material in its original Spanish form, along with accompanying English translations; see the “Supporting Information” section at the end of this article.)

To examine whether the consent form length was related to mean correct responses or willingness to participate, we ran a two‐sample independent t‐test and a Wilcoxon rank‐sum test, respectively. Percentages provided for quantitative results may occasionally not sum to 100% due to missing values.

## Interview Findings: Comprehension of Consent Materials

**O**verall, comprehension as indicated by performance on the 15‐item quiz was high (see table 2 for complete quiz results). The average number of items answered correctly was 13 (range: 10 to 15), and there was no statistically significant difference by consent form (p = 0.31). The mean number answered correctly among those who read the traditional form was 13.3 (range: 11 to 15), and among those who read the simplified form, it was 12.8 (range: 10 to 15). The items most commonly answered correctly related to blood draws, profit sharing, expectations for individual research results, and the right to withdraw from the study. The items most often answered incorrectly were about the provision of medical care, access to medical records, notification when samples or data are used, large‐scale data sharing, personal health benefits, and the purpose of the project.

Several participants who incorrectly answered the question regarding personal benefits insisted that the possibility of receiving results relevant to one’s health (in the event a serious and medically actionable condition was discovered) was a clear benefit. As one person simply put it, “The benefit to your health would be to be told, ‘Look, you have something’” (LA, 18, traditional).

## Reassuring Consent Form Information

**R**egardless of which consent form they read (traditional or simplified), participants tended to find the same types of information reassuring. Most mentioned being at least somewhat reassured by federal protections to guard against employment and health insurance discrimination, and about three‐fourths were reassured that participation in the study was voluntary and that they could withdraw at any time. As one put it, “They are letting you know that if you want you can join the study or not, and that if you join and one day say, ‘I don’t want to be a part anymore,’ then you can quit. I like it—I like it a lot, it’s very important” (LA, 04, traditional).

About half felt reassured by mechanisms and procedures to protect information, such as passwords, encryption, and physical security measures. Among those participants, many appreciated that the biobank would get permission before sharing identifying information with others and felt especially reassured by the use of codes to replace participants’ names.

One participant said,

Well, this kind of helps with the previous part [describing privacy risks]; it’s like you get scared, and then you get all the information we need to calm down again: that [the data are stored in a] building apart from where the medical records are—that is huge, gives me lots of peace of mind, because it means that it’s its own thing, with its own protection, and that they change the specific information with the number [code], and that people, the researchers, don’t know our name, nothing; this makes one feel very protected. So, I like it. (NY, 08, traditional)

Some participants felt comforted that researchers’ applications to study the stored specimens and data would undergo scientific and ethical review, associating the review processes with professionalism, legitimacy, and formality. As one person explained, “If you look at it, it is very professional, very legal too” (NY, 04, simplified). Others were reassured by the transparency with which risks were discussed. “They tried to be as transparent as they could be,” one observed. “They did not mention that, ‘Oh, you do not have to worry; nothing bad will happen.’ They mentioned the possibility that in the future a more sophisticated, easier way to obtain information from a specific person may be invented. What caught my attention was that they did not deny the possibility that this could happen in the future; they let you know” (NY, 01, simplified). Finally, several interviewees felt reassured that there were limits on the number of times they would be contacted to participate in other research and that, even if contacted, they still had the right to decline. “[I like that participating in other studies is] voluntary—you can decide to participate or not,” one person said, “and that they won’t contact you about more than two studies a year, which is also excellent, because you don’t want for them to be calling you every week” (NY, 06, simplified).

## Concerning or Confusing Consent Form Information

**D**espite finding several aspects reassuring, participants found other information in the consent form concerning or confusing. Most issues pertained both to the traditional and simplified forms, but, as noted, a few were specific to one form.

### Data sharing

Concerns regarding data use were common, regardless of which form participants received. A few wanted additional information about the biobank review process, including *how* decisions would be made and how their data could be used. As one person explained, “[The form] is saying that they will give [biobank data] to who they want to, so it’s like a huge network. It’s a little like, I don’t know, like, who’s deciding that?” (NY, 08, traditional).

More often, participants had questions about *who* could access their data and about the motives, security capabilities, and trustworthiness of these individuals to protect and use data appropriately. As one asked, “The people who are going to have this access to my information, how can I trust them?” (LA, 03, simplified).

Some were especially concerned about commercial companies, government entities, and international researchers having access to their information, as these quotations indicate:

The government is very big, and you never know how it’ll use it, right? (LA, 02, simplified)

I like the part of universities; I like everything that has to do with the public service because they are studies, but the pharmaceutical companies I don’t like because then we are talking about a private company; they can profit. (LA, 19, simplified)

Well, when you think the data are going to be researched not only in the United States but in other countries, you say, “Okay, well, in this country I can guarantee certain things, but outside of it, I do not know what can happen.” That is something that can cause concern. (LA, 01, traditional)

### Genetic Information Nondiscrimination Act

Regardless of which form they read, many participants found information about the Genetic Information Nondiscrimination Act (GINA) to be confusing. Most often, they were confused or concerned by the discussion of life and disability insurance. Some failed to see any connection between biobank participation and insurance and thus did not understand *why* insurance companies would even be mentioned. As one person said, “Okay, in this section I don’t get what life or disability insurance has to do with this project. What do they have to do with the study?” (LA, 03, simplified). Some—particularly those who read the traditional form—found information regarding GINA to be contradictory. Specifically, they thought the statement describing GINA’s inability to protect against life and disability insurance discrimination contradicted previous assurances that information would not be shared with insurance companies. For example, a participant stated, “Yeah, I’m a little bit confused with the last section because it says, ‘GINA will not protect you against genetic discrimination from companies selling life insurance.’ I don’t get it because it says we will protect you, but later it says that we won’t. That confused me a bit” (LA, 04, traditional). And another expressed, “This alarms me because why are you going to tell me this when before you told me that my information wasn’t going to be exposed to insurance companies?” (LA, 01, traditional). Participants also questioned *how* insurance companies would be able to access their information. As one put it, “GINA will not protect me against life or disability insurance companies. This worries me, because how can they access that information?” (LA, 08, traditional).

### Medical records access

As illustrated by the following quotations, some participants were also confused about *how* researchers would gain access to medical records and *who* would provide researchers with authorization to view them:

What leaves me a bit confused is this that says, “We will get information from your medical records, such as test results, medical procedures, X‐ray images, the medicine you take.” So, how would they do that? Would they be contacting my doctor? Are they contacting my health insurance? (NY, 14, simplified)

It says, “We’ll get information from your medical records.” Is that information I provide, or will they contact the doctors? (NY, 06, simplified)

I guess [my medical record is] in a national database, right? (NY, 11, simplified)

Confusion regarding how and from whom researchers would access information seemed primarily limited to participants who read the simplified form. This may be because the traditional form stated that “we will collect some information from your medical records *at Duke*” (emphasis added), while the simplified form did not specify from where medical records information would be collected.

Language in the traditional form also caused some confusion, particularly related to the biobank’s role in providing medical care. The form stated, “Second, we will collect some information from your medical records at Duke. Examples include information about lab results, medical procedures, images (such as x‐rays), and medications. This is because future researchers need to know if you have any health problems. They may also need to know about any treatments you have had and how well the treatments worked. We will look at your medical record from time to time to update this information.” Part of this disclosure was intended to convey *why* researchers need access to medical records (to obtain clinical data to correlate with their analysis of biospecimens), which was unclear to a few readers of the simplified form, such as the one who asked, “[Why are x‐rays needed?] For studies? For research? To confirm that the person is sick? It doesn’t say specifically the reason that x‐rays will be needed” (NY, 09, simplified).

However, a few interpreted the additional explanation included in the traditional form to suggest either that future researchers would review a patient’s images or results to make clinical diagnoses *for* the patient or that researchers *themselves* would be updating the patient’s medical record with new information relevant to the patient’s care. For example, one participant said, “It seems to me that as they collect the medical information, and if I have something, they will contact me” (LA, 08, traditional), and another stated, “Well, it would give me peace of mind if they find anything. Something that is affecting my health and I do not know. That they would contact my regular doctor” (LA, 18, traditional).

### Certificate of Confidentiality

In both the traditional and simplified forms, language describing the Certificate of Confidentiality caused confusion among participants. Among those who read the simplified form, this confusion related to a specific sentence. The English phrase “to fight any legal demand” was mistakenly translated verbatim to Spanish, which led a few participants to question whether this certificate was intended to fight *for* or *against* any legal demand. One person observed, “It does not make it very clear to know if this [certificate] is good or bad or how you are benefiting—like it is not very clear” (NY, 09, simplified). For the sentence to have had its intended protective meaning, the preposition “against” should have been included in the Spanish translation (to fight *against* any legal demand).

Among those who read the traditional form, nearly half found the certificates section confusing, but for a different reason. Most understood that a certificate protects data against compelled disclosure in a legal proceeding; however, they were confused by the exceptions (such as mandatory public health reporting) and found the language contradictory, as these statements indicate:

I’m a little confused because it talks about how they can’t hand over information even at the request of a judge. But in another part it also says that they are obligated to give the information to the government when they ask for it. So I kind of don’t understand how they are—what do they base it on if they decide to do it or not? (NY, 02, traditional)

This part has me a little confused … I’m not sure whether they do or do not share information with the government. I don’t understand this a bit. (NY, 05, traditional)

One person found the language so contradictory that he assumed it was a translation error, remarking,

It says, ‘In addition, we will disclose information about you without your consent,’ […]—it should be, “In addition, we will *not* disclose your information without your consent.’ I don’t know, maybe, you have to be clearer in this part.” (LA, 18, traditional)

Finally, a few simply did not understand why information regarding exceptions was relevant or how it related to biobank participation. Along these lines, one participant said, “If you discover that there’s domestic violence, or abuse of someone, I don’t understand how. How would you know if there is domestic violence through the blood?” (LA, 16, traditional).

### Description of quantity of blood to be drawn

Regardless of which form participants received, about one‐third of them took issue with using tablespoons to quantify the amount of blood to be drawn (reacting to the language “We will use a needle to draw about three tablespoons of blood from your arm.” Most associated “tablespoons” with cooking; thus, as the following responses show, using it to refer to a blood sample seemed inappropriate and out of context, and for some participants, it conjured up strange images:

“Spoonful” doesn’t seem very appropriate. Because the spoon is related to food, no? So no one eats blood. (LA, 17, simplified)

It sounds weird. I wouldn’t know how to give an example of what you could put instead, but three tablespoons sounds like you’re going to get the spoon out and they’re going to put it there so that you bleed [onto the spoon]. I mean, I don’t know. (NY, 07, traditional)

It sounds more like food—like you’re cooking a recipe. (LA, 05, traditional)

While several thought that providing the quantity in milliliters was sufficient, others suggested referencing the number of vials or tubes to be collected, and one participant proposed rephrasing the sentence to read “the equivalent of about three tablespoons of blood” (LA, 19, simplified).

### Concerns about tone and formality

Participants raised issues about tone and formality regardless of which consent form they read. More than one‐fourth identified phrasing they felt evoked a degree of casualness or lack of professionalism that they perceived to be at odds with the type of document and research endeavor under consideration. Participants most often took issue with the term “promise” (*“promesa”*), which was used to describe researchers’ agreement to not reidentify participants and to keep coded materials secure. Many described the term as too informal, childish, and lacking the appropriate formality or enforceability necessary for this type of study. Participants suggested replacing the term with words like “privacy policy” (*“póliza de privacidad”*) or “signed contract” (*“contrato firmado”*). Specific reactions to the term included the following:

Promises are never … it’s like, no, that word isn’t used for that. I can’t think of the appropriate word now—but it would have to be some more legalistic term. (NY, 07, traditional)

No, I don’t like “promise.” It’d be more like a “privacy policy.” That’s what you are trying to say, right? (LA, 14, traditional)

It sounds like I’m reading a story to a five‐year‐old—a promise. The word “promise” does not give me a sense of security. In fact, it could be even best to get rid of this phrase … . [If it said] “under a signed contract” that would be different, not with a promise. (LA, 06, simplified)

In another critique of tone and formality, a few participants took issue with the phrase “time to time” in the sentence “We will use your medical record from time to time (*“de vez en cuando”*) to update this information,” perceiving it to be too informal for a consent form and suggesting the need for a more “professional” (*“profesional”*) word, such as “periodically” (*“periódicamente”*) (NY, 02, traditional).

Participants also felt some language communicated a standoffish, defensive, or aggressive tone. As one example, a few suggested the statement “you will be contacted no more than once a year” was too negative. One said, “Yeah, this business that we will contact you ‘no more’—this of ‘no more,’ you use it too much. I think it is negative” (NY, 03, simplified). And another recommended, “There has to be a way of softening this a bit. I think that this line, ‘We will contact you no more than once a year to use this information,’ you use a negative, not a positive [way] of communicating the information. It [should be], ‘In the future, we will contact you once a year to update this information.’ Sure. Stating ‘no more’ already indicates a bit of a defensive[ness]” (LA, 05, simplified).

Participants also suggested a range of revisions that would have the effect of downplaying the risk. Several felt strongly that the consent form should describe protections before risks to avoid causing anxiety. As one interviewee suggested, “I would put this part about risks later because you’re always telling me about risks first, and you’re not showing all that you’ll do to protect my data. I’m worried in this regard about the order in which this information is presented… . [You should put that phrase] in all caps so that you highlight ‘WE ARE GOING TO DO EVERYTHING TO PROTECT YOU IN ANY WAY WE CAN’” (LA, 14, traditional).

Other commonly proposed changes included the use of passive voice constructions and different adjectives to soften language and convey less risk. For example, some participants commented on the sentence “We believe the chance [that someone will get access to the data we have stored about you] is very small, but we cannot make guarantees.” Some took issue with the phrase “very small” (“*muy pequeño*”), all suggesting we replace it with “minimal” (*“minima”*) because they felt “minimal” denoted an even smaller risk.

Several others found the phrase “we cannot make guarantees” (“*no podemos garantizarlo*”) to be too forceful and negative and suggested alternative, passively constructed phrasing that would rhetorically distance the biobank from the inability to guarantee protections. One person explained, “When it says, ‘*We* cannot [guarantee],’ it’s like they are leaving you all alone. I think that it should say, “*There are no* guarantees’” (LA, 05, traditional).

Some participants also recommended revisions to language describing potential benefits. Several were dissatisfied with the phrase “You should not expect to get direct health benefits” (*“No debe esperar obtener beneficios directos para su salud”*) in the traditional form, and “You will not get direct benefit” (*“Usted no obtendrá un beneficio directo”*) in the simplified form, arguing that the statements were too frank and negative. Some suggested replacing active constructions with passive voice to soften the effect. One advised, for instance, “The phrases you use [referring to ‘you should not expect’], it can be that no—they can even be a bit aggressive. Instead of saying, ‘You shouldn’t expect,’ simply say, ‘There are no direct benefits’” (NY, 02, traditional).

A few others recommended, as in the following examples, that the section be reorganized to highlight societal benefits and minimize—or remove any mention of—the lack of personal benefits:

Well, I understand what the project is about and all that, but still, as a human being, it’d be nice maybe if there was some benefit, not that the first thing says, “You will not obtain any direct benefit.” Maybe that can go at the end. First, maybe it could read something like, “The benefit is great because it is a benefit in the future for humanity, for our kids,” maybe starting this section like this. And then at the end perhaps adding, “No direct benefits will be obtained from participating.” (LA, 06, simplified)

“The main benefit is that you wish to participate and collaborate in research and reach discoveries that could benefit others in the future. There is no monetary reward.” And there, leave it like that. Just don’t write that there is no benefit. (LA, 14, traditional)

## Amount of Information and Willingness to Participate

**W**e also asked participants to rate their satisfaction with the amount of information provided and their willingness to participate in the hypothetical biobank. Few participants thought their form contained too little information. However, over 40% of those who received the traditional form felt it contained too much information, compared to 15% of those who received the simplified form (see table 3). Three‐fourths of those who received the simplified form perceived it to contain the “right” amount of information, compared to fewer than half of those who read the traditional form. All these results are consistent with those found in English‐speaking populations.

When asked at the end of the interview about their willingness to participate in the hypothetical biobank, nearly three‐fourths said they probably or definitely would take part, often citing potential public health benefits, altruism, and perceived low risk as reasons to participate (see table 4). Most of those who said they probably or definitely would not participate did not specify a reason, but a few cited a fear of needles and a desire to speak with someone about the study. More than one‐third of those who received the simplified form said they probably or definitely would not participate, compared to only about 10% of those who received the traditional form.^22^

## Discussion

**A**lthough English‐language consent forms are routinely translated into Spanish, cognitive testing is not necessarily conducted to ensure the accuracy, comprehensibility, and cultural appropriateness of materials provided to Spanish‐speaking populations. In this study, we tested two rigorously developed Spanish‐language consent forms to assess comprehension, satisfaction with the amount of information, and hypothetical willingness to participate, and to examine elements participants found reassuring, concerning, or confusing.

Comprehension did not differ by form, a finding consistent with other studies involving native English speakers.^23^ In general, comprehension was high; however, a notable minority failed to answer questions about key aspects of the hypothetical biobank correctly. Misconceptions regarding the personal health benefits of participation and provision of health care are particularly concerning, as these may motivate participation. Multiple factors, including participants’ assumptions regarding researchers’ ethical obligations to return results,^24^ as well as specific language in the traditional consent form, may have contributed. Additionally, participants appear to have focused on different sections of the consent form to arrive at their understandings: some frequently referred to a sentence noting the small chance of learning something relevant to one’s own health while glossing over two other sentences stating that they should not expect individual results or health benefits. Moreover, while a few participants may have overestimated the likelihood of receiving results, others simply had the perspective that, however unlikely, *any* chance of receiving results is a benefit.

Although improvements to our translations of the consent forms may help correct some of these misunderstandings, participants’ expectation that they will be notified if researchers find something serious about their health has also been noted in studies conducted with English‐language speakers.^25^ Evidence suggests that, even when consent materials use very conservative language to caution participants not to expect results, a fair number still believe that researchers will find a way to communicate the information to them.^26^ This belief is particularly problematic if participants misinterpret the lack of results as meaning they have no serious health problems (“no news is good news”).^27^ Our findings suggest that attention is needed to ensure that consent materials emphasize the research purpose of biobanks and clearly distinguish between the roles of researchers and physicians.^28^

Some of the concerns and confusion voiced by participants arose more often depending on the form. Those who received the traditional form, for instance, more often perceived specific language to be contradictory (for example, concerning the scope and limits of individual protections). These findings suggest there is a fine line between providing exhaustive detail in the interest of being thorough and overwhelming the primary intended message about the overall level of risk and protections, leading to reduced comprehension and confusion.^29^

Regardless of which consent form participants received, they voiced concerns about data use and data sharing. Most often, they wanted more information regarding how their medical records would be accessed, who might use their data and for what purposes, and the review processes for making those determinations. These findings are consistent with studies among English‐language speakers^30^ and may point to the need to provide participants with examples of possible users and uses of data, as well as information regarding governance and oversight.

Although participants were concerned by some aspects of the biobank, many were simultaneously reassured by the transparency with which risks were disclosed, and interpreted this as a sign of trustworthiness. The value English‐speaking participants place on transparency in biobanking has been well documented.^31^ Crafting clear disclosures—especially about aspects of biobank participation that people may find risky, concerning, unexpected, or unfavorable (such as commercial use of data and public data sharing)—helps promote transparency while providing researchers with an opportunity to build trust with prospective participants.^32^

Many issues identified through our interviews have also been documented with English‐speaking participants, though other issues may be more specific to the translation process or to Spanish‐speaking populations. Qualitative data captured through cognitive interviews allowed us to identify subtle but important issues with the materials as translated to Spanish. In one case described above, an incorrect translation (that is, a failure to add a necessary preposition to the Spanish document) left opaque the essential meaning of language describing certificates of confidentiality. In another case described earlier, participants understood the basic meaning of a sentence about having blood drawn, but they found the specific reference to tablespoons to be awkward.

Cognitive testing also allowed us to identify potential issues with tone, formality, and voice. Participants felt some consent language was too abrasive, while other language was too informal for the project under consideration. They also suggested changing several active‐voice constructions to the passive voice, particularly in cases referencing the researcher or participant. These kinds of concerns may reflect fundamental differences in English and Spanish speakers’ expectations regarding professional documents.^33^ In English academic writing, for example, key words and phrases are often repeated to enhance cohesion and reinforce logical connections, while in Spanish, value is placed on an “elaborate style of multiple clauses, an elevated lexicon, deliberate deviation from the stated topic, and fewer direct coherence progressions.”^34^ Linguists have described English as a writer‐responsible style,^35^ in which texts are written to be explicit and “anticipate and eliminate any difficulties that the reader may have in understanding [the] text.”^36^ Spanish, on the other hand, is a reader‐responsible style, with the reader assumed to be “an intelligent being ‘to whom very little needs to be explained.’”^37^

Many of these characteristics of written Spanish are seemingly at odds with recommended actions to simplify consent forms. Indeed, to increase comprehensibility and clarity, it is widely recommended that consent forms be written in active voice, use simple sentence structures, and avoid complex, technical, or overly formal language.^38^ These recommendations may align more closely with a reader‐responsible style of writing and thus be more likely to achieve their intended goal in English‐language materials. Although we cannot assign cause and effect, it is nonetheless interesting to observe that our participants who received the simplified form indicated significantly less willingness to participate—despite much higher likelihood of saying they had received the right amount of information and no differences in comprehension quiz scores (compared to those who received the traditional form). Spanish speakers may understand the core meaning expressed using active voice and simple, informal words and sentences, but important pragmatic meaning—as well as the expected style and tone (for example, formal, polite, indirect)—may be or is lost. As Montaño‐Harmon cautions, if used in Spanish writing, the “deductive, linear discourse pattern deemed logical and organized in American English” would sound “simplistic and juvenile” and could “project a hidden message of abruptness, even rudeness, insulting [a] Spanish‐speaking reader.”^39^

It is essential to identify the kinds of translation‐related issues we encountered, although some—such as the inadvertent omission of a required word—may be easier to address than others. Revisions related to tone, formality, and voice should be carefully considered to ensure that any changes to improve cultural congruence do not come at the expense of comprehension, clarity, or accuracy and that revisions continue to meet regulatory requirements and best practice guidelines.^40^

To the extent that a research endeavor is committed to enrolling participants who speak limited to no English, commensurate resources are needed to ensure quality translation of informed consent materials. Elsewhere we have outlined a series of steps to improve the translation process, including—when feasible—working with interpreters who are fluent in both languages and cultures *and* who are familiar with the goals for the ethical conduct of research, encouraging the research team to take an active role in the translation process, producing a back translation and comparing it to the original English‐language materials, and developing a systematic process to compare forward and back translations and make linguistically and culturally appropriate revisions.^41^ Yet as our present results illustrate, even consent materials translated using a highly rigorous process should be cognitively tested to ensure accuracy, comprehensibility, and cultural appropriateness.

Our study has several important strengths. To our knowledge, ours is one of few studies^42^ to conduct cognitive testing to examine how native Spanish speakers interpret specific concepts and language in translated consent forms. Our sample included a cross‐section of 39 participants speaking 12 dialects of Spanish; thus, we likely captured a diverse range of regionally specific translation issues. The combined use of open‐ended qualitative questions and a comprehension quiz allowed us to explore subjective interpretations of consent form information and perceptions of length while also assessing objective comprehension.

Our study is nevertheless subject to several limitations. First, participants were asked to review consent materials for a hypothetical biobank; we cannot be certain whether individuals presented with an actual biobank research opportunity would have identified the same concerns, weighed concerns differently, or sought different types of information. In addition, although our sample size included broad demographic diversity, our findings are limited to a relatively small sample of individuals in two large metropolitan areas. Future research should continue to explore issues related to tone, formality, and voice and to examine whether and how widely accepted recommendations for simplifying English consent forms are suitable for Spanish‐language materials. Such studies should capitalize on the benefits of cognitively testing translations to improve the accuracy, comprehensibility, and cultural appropriateness of consent materials intended to increase participation by populations typically underrepresented in biomedical research

## SUPPORTING INFORMATION—TABLES AND SUPPLEMENT

The four tables and the supplement containing the Spanish language and English translations are available in the “Supporting Information” section for the online version of this article and via *Ethics & Human Research*’s “Supporting Information” page on The Hastings Center’s website: https://www.thehastingscenter.org/supporting-information-ehr/.

## Acknowledgments

Thanks to our colleagues Zachary Lampron and Colin Halverson for their assistance.

## Supporting information

Additional supporting information may be found in the online version of this article:

Supporting Information
