# Supplementary material for: Improving Translation and Cultural Appropriateness of Spanish‐Language Consent Materials for Biobanks
Source: Ethics Hum Res. 2019 Sep 20;41(5):16–27. doi: 10.1002/eahr.500028 (PMC6856797; doi:10.1002/eahr.500028)
Supplement: Supplementary file 1 — Supporting Information [file EAHR-41-16-s001.pdf]

Improving Translation and Cultural Appropriateness of Spanish-Language Consent Materials for Biobanks

Kathleen M. Brelsford, Ernesto Ruiz, Catherine M. Hammack, and
Laura M. Beskow

Table 1.
Participant Characteristics

	Total *n (%)*	*Traditional form* *n (%)*	*Simplified form* *n (%)*
Total participants	39	19 (49)	20 (51)
Gender			
Men	17 (44)	9 (47)	8 (40)
Women	22 (56)	10 (53)	12 (60)
Site			
Los Angeles	19 (49)	10 (53)	9 (45)
New York City	20 (51)	9 (47)	11 (55)
Age group			
≤ 34	12 (31)	8 (42)	4 (20)
35-44	15 (38)	5 (26)	10 (50)
45+	12 (31)	6 (32)	6 (30)
Education			
≤ high school	6 (15)	3 (16)	3 (15)
some college	20 (51)	10 (53)	10 (50)
bachelor's degree	11 (28)	5 (26)	6 (30)
postgraduate education	2 (5)	1 (5)	1 (5)
Place of origin			
Argentina	4 (10)	1 (5)	3 (15)
Colombia	5 (13)	2 (11)	3 (15)
Cuba	2 (5)	1 (5)	1 (5)
Dominican Republic	7 (18)	4 (21)	3 (15)
Ecuador	2 (5)	1 (5)	1 (5)
Guatemala	1 (3)	0 (0)	1 (5)
Honduras	1 (3)	1 (5)	0 (0)
Mexico	6 (15)	3 (16)	3 (15)
Nicaragua	2 (5)	1 (5)	1 (5)
Peru	4 (10)	2 (11)	2 (10)
Puerto Rico	4 (10)	2 (11)	2 (10)
Venezuela	1 (3)	1 (5)	0 (0)

Table 2.
Performance on Comprehension Assessment

Participants who answered the following correctly	*Total (n = 39)* *n (%)*	*Traditional form (n = 20)* *n (%)*	*Simplified form (n = 19)* *n (%)*
Q1. What is the purpose of this project? [correct: collect and store samples and health information for future research]	33 (85)	16 (84)	17 (85)
Q2. Which of the following is something the biobank will NOT do? [correct: provide me with medical care]	30 (77)	15 (79)	15 (75)
Q3. T/F: You will have blood drawn as part of this project. [correct: true]	39 (100)	19 (100)	20 (100)
Q4. T/F: The biobank will collect information from your medical records. [correct: true]	28 (72)	13 (68)	15 (75)
Q5. T/F: The biobank will let researchers study your samples and information. [correct: true]	36 (92)	18 (95)	19 (95)
Q6. Someone from the biobank might contact you about all of the following EXCEPT [correct: to let me know every time my samples and information are used in a study]	26 (67)	13 (68)	13 (65)
Q7. T/F: If you participate in the biobank, you will get personal health benefits from the research. [correct: false]	33 (85)	18 (95)	15 (75)
Q8. T/F: Unless you decide to stop taking part, there is no limit on the length of time the biobank will keep your samples and information. [correct: true]	36 (92)	19 (100)	17 (85)
Q9. T/F: Some of your genetic and health information might be put into a database outside the biobank. [correct: true]	24 (62)	12 (63)	12 (60)
Q10. The chance that someone could get access to your information or trace it back to you is ____ [correct: small, because the biobank will take many steps to protect my privacy]	35 (90)	18 (95)	17 (85)
Q11. T/F: The biobank will give researchers information that easily identifies you. [correct: false]	36 (92)	17 (89)	19 (95)
Q12. T/F: There are no laws to protect against discrimination based on your information. [correct: false]	36 (92)	18 (95)	18 (90)
Q13. If research using your sample helps lead to any new products or treatments ____ [correct: I will not get any of the profits]	39 (100)	19 (100)	20 (100)
Q14. If the researchers use your sample and information in a study ____ [correct: I should not expect to get my individual results]	39 (100)	19 (100)	20 (100)
Q15. Once you sign up for the biobank, can you change your mind? [correct: yes, but the biobank cannot get back samples and information from studies that have already begun]	38 (97)	19 (100)	19 (95)

Table 3.
Amount of Information Provided

	Total (n = 39)	Traditional form (n = 19) n (%)	Simplified form (n = 20) n (%)	P-value n (%)
Amount of Information				0.03
Way too little	2 (5)	1 (5)	1 (5)	
Too little 2 (5)	1 (5)	1	(5)	
About right	24 (62)	9 (47)	15 (75)	
Too much9 (23)	6 (32)	3	(15)	
Way too much	2 (5)	2 (11)	0 (0)	

Table 4.
Willingness to Participate in Hypothetical Biobank

	Total (n = 39) n (%)	Traditional form (n = 19) n (%)	Simplified form (n = 20) n (%)	P-value
Definitely willing	12 (31)	9 (47)	3 (15)	0.02
Probably willing	16 (41)	7 (37)	9 (45)	
Probably not willing	6 (15)	1 (5)	5 (25)	
Definitely not willing	3 (8)	1 (5)	2 (10)	
